# Nestin in multiple myeloma: emerging insights into a potential therapeutic target

**DOI:** 10.3389/fonc.2025.1596928

**Published:** 2025-07-29

**Authors:** Yingmiao Wu, Ji Luo, Yue Zhou, Jiaoya Lin, Yajie Wu, Shuai Zheng, Jiao Chen, Feifei Che, Qiang Wang, Ling Zhong

**Affiliations:** ^1^ Genetic Diseases Key Laboratory of Sichuan Province, Department of Medical Genetics, Department of Laboratory Medicine, Sichuan Academy of Medical Sciences & Sichuan Provincial People’s Hospital, School of Medicine, University of Electronic Science and Technology of China, Chengdu, Sichuan, China; ^2^ School of Medicine, University of Electronic Science and Technology of China, Chengdu, Sichuan, China; ^3^ Department of Hematology, Sichuan Provincial People’s Hospital, University of Electronic Science and Technology of China, Chinese Academy of Sciences Sichuan Translational Medicine Research Hospital, Chengdu, Sichuan, China; ^4^ Center for Translational Research in Hematological Malignancies, Houston Methodist Neal Cancer Center, Houston Methodist Research Institute, Houston, TX, United States

**Keywords:** nestin, multiple myeloma, multiple myeloma stem cells, cancer stem cells, therapeutic target

## Abstract

Multiple myeloma (MM) is the second most common hematological malignancy and remains incurable, with high rates of relapses and refractory. One of the root causes is the presence of multiple myeloma stem cells (MMSCs). The deficiency of MMSC treatment lies in the lack of specific targets. CD19, CD138, CD27, and ALDH have been regarded as markers for MMSCs; however, none of them can reliably identify MMSCs. Therefore, identifying unique markers of MMSCs is crucial. Nestin, a class-VI intermediate filament protein, was originally described as a marker of neuroepithelial stem/progenitor cells. Recently, nestin has been reported to be a useful marker and therapeutic target of cancer stem cell (CSC) in solid tumors, reflecting its importance in drug resistance and poor prognosis. Although nestin has been reported to be associated with poor prognosis in MM, its biological role in MM has not yet been thoroughly explored. This review summarizes the latest research progress of nestin in MM, including the characteristics of nestin, its role in CSCs across different cancers, the current status and cutting-edge detection technologies of MMSC, involved signaling pathways and clinical relevance in MM. It emphasizes that nestin is a more specific and effective potential therapeutic target for MMSC.

## Introduction

Multiple myeloma (MM) is the second-most common hematological malignancy, accounting for 10-15% of all blood cancers (1). Although standard first-line treatments including proteasome inhibitors, immunomodulators, and dexamethasone, as well as novel CAR-T cell therapies have significantly improved efficacy, MM remains incurable, with frequent relapse and drug resistance (2). One crucial reason originates from multiple myeloma stem cells (MMSCs). MMSCs represent a distinct subpopulation of tumor-initiating cells characterized by self-renewal capacity, multilineage differentiation potential, tumorigenicity, and drug resistance, which are considered the key determinants of disease relapse and treatment refractoriness. MMSC not only have the potential to induce malignant tumors, but also exhibit invasion, metastasis, and resistance to radiotherapy and chemotherapy (3). CD19, CD138, CD27, and ALDH have been regarded as markers for MMSCs. Unfortunately, despite the availability of specific targeted and highly sensitive detection methods, precise markers for MMSC are still lacking, resulting in very limited treatment for it. Therefore, it is essential to discover specific target for MMSC.

Nestin, a class-VI intermediate filament protein, was originally described as a marker of neuroepithelial stem/progenitor cells in the central nervous system. An increasing number of studies suggest that nestin is involved in the regulation of proliferation, invasion, and drug resistance in various cancers. Additionally, nestin has already been recognized as a specific stem cell marker in certain cancers, including brain (4), pancreatic (5), prostate (6), and bladder cancers (7), contributing to poor prognosis and outcomes (8).

This review summarizes nestin as a potential therapeutic target for MMSCs. Firstly, we described the characteristics of nestin, and its role in CSCs. Subsequently, the advantages and disadvantages of current MMSC markers and the challenges of detection technologies were summarized. Finally, we explored the relationship between nestin and MM, including signal pathways, treatment, clinical relevance, and potential directions in future research. In all, the review’s synthesis of current knowledge underscores the clinical relevance of nestin and highlights important areas for future research, not only as a prognostic indicator but also as a potential target for innovative therapies aimed at overcoming treatment resistance in MM.

## Characteristics of nestin

**Figure 1:**
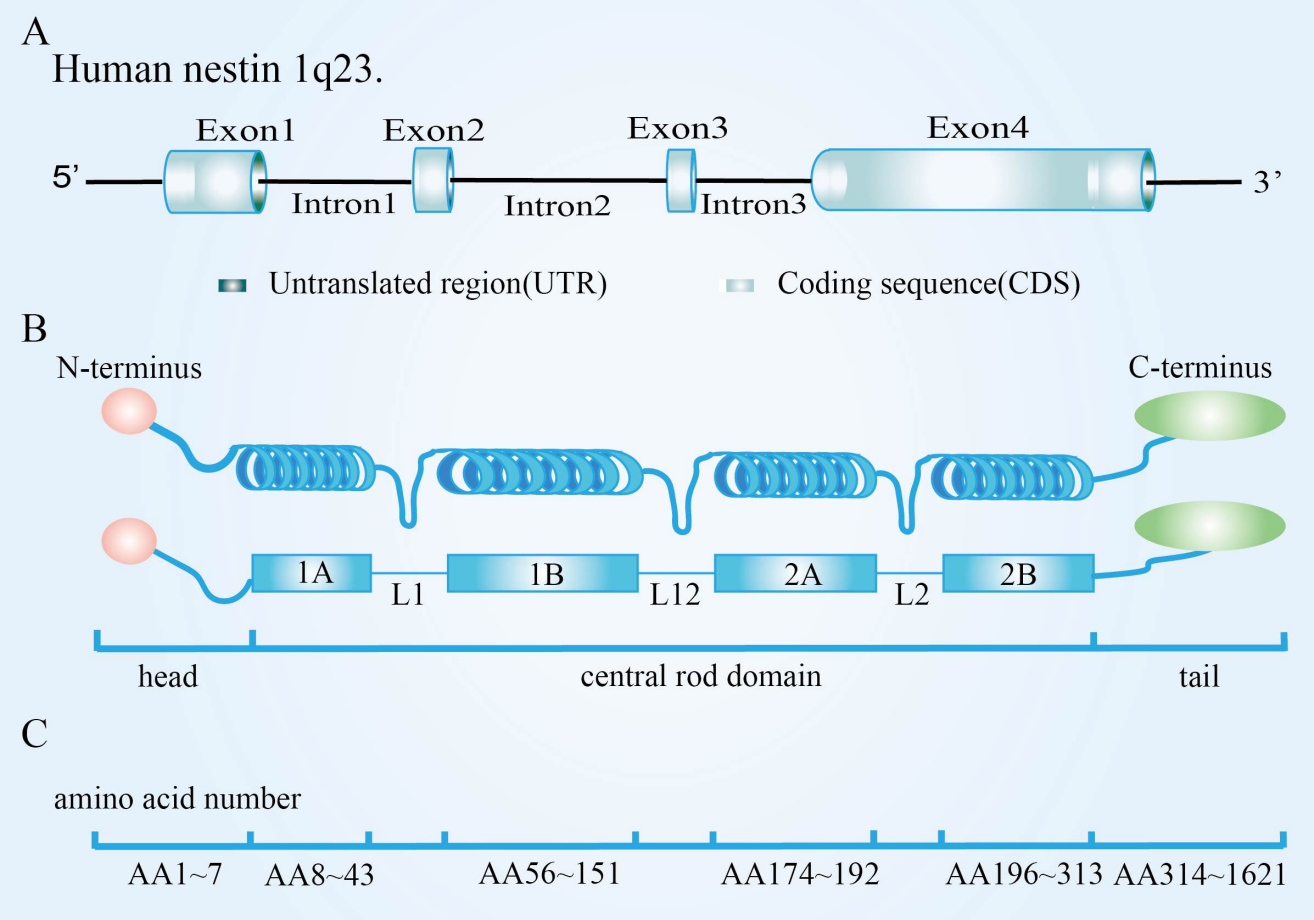
Nestin structure and assembly into intermediate filament. **(A)** Molecular structure of nestin; **(B)** Particularities of nestin monomer; **(C)** Nestin forms heterodimers. Adapted from Bernal et al, 2018. Originally published in Cellular and Molecular Life Sciences, 75(12):2177-2195. Copyright Springer Nature Publishing. Adapted with permission (11).

Nestin is a class VI intermediate filament protein consisting of an N-terminal rod domain and 41 heptapeptide repeats in the C-terminal region. Originally found in human central nervous system (CNS) tumors, it was expressed primarily in CNS CSC in rats (9), described as a marker of neuroepithelial stem/progenitor cells in the CNSs of rats (10). The human nestin gene is located at 1q23.1, composed of four exons separated by three introns, two of which shared with neurofilaments, suggesting the possibility that nestin and neurofilaments are descended from a single ancestor. Its promoter is located in the 5’ untranslated region, containing two Sp-1 binding sites but lacking a functional TATA box. Enhancing elements were in the first and second introns, with enhancers in the first specifically designed to increase the nestin expression in myogenic precursors; The second intron contained two neuroprecursor-specific enhancers (**Figures 1A, B**) (11). Human nestin is composed of 1621 amino acids, but the nestin N terminal is short, composed of only 11 amino acids. Further, the C-terminal is long, with 1,479 amino acids (**Figure 1C**), and it contains many charged amino acid repeats, usually in two forms: the 220kDa glycosylated form and the 177kDa de-glycosylated form, though the former is more common (12). Nestin does not readily fold into its functional conformation independently. It requires the presence of another copolymer, such as vimentin. Because of the characteristic core domain, the presence of a third intron, the short head domain, and the unconventionally long C-terminal end, nestin is regarded as a separate type-VI IF (13). Furthermore, we cannot ignore that nestin protein is a dynamic structure whose phosphorylation/dephosphorylation modulates the disassembly and assembly of intermediate filaments and whose C-terminal domain interacts with microtubules and microfilaments (14). These mechanisms might play a role in the rapid redistribution of intracellular proteins during key cell processes (15–17).

## The role of nestin in CSCs across different cancers

Cancer stem cells (CSCs) are a rare population of cells within tumor tissue that possess the ability to self-renew and differentiate into various cancer cell precursors. CSCs are crucial in tumor recurrence, metastasis, and heterogeneity (18). A multitude of studies have discovered that neural stem cell marker-nestin plays a critical role in regulating CSCs, and closely associates with poorer tumor grading and prognosis.

A large number of studies have shown that the overexpression of nestin can regulate the stemness to enhance proliferation and migration in glioblastoma (19), lung adenocarcinoma (20), colorectal cancer (21), pancreatic cancer (22), triple negative breast cancer (23) and cervical cancer (24). Knockdown of nestin expression significantly inhibit the proliferation and colony formation of non-small cell lung cancer, leading to G_1_ phase of the cell cycle and inhibiting AKT activation (25). Reducing the phosphorylation of nestin can inhibit the proliferation (26). Nestin accelerates invasion by promoting EMT (epithelial-mesenchymal transition). Nestin induces the expression of N-cadherin, thereby promoting EMT in breast cancer and pancreatic ductal carcinoma (27, 28). Nestin is partially co-localized with CD56 and vimentin, participating in EMT in cHCC-CCA (29). Nestin promotes the expression, activation, and nuclear translocation of β-catenin, leading to EMT in liver cancer cells.

Furthermore, nestin is also associated with drug resistance. Nestin competes with Nrf2 for binding to Keap1, thereby promoting the production of antioxidant enzymes, enhancing the antioxidant stress resistance in non-small cell lung cancer and gastric cancer, and consequently contributing to drug resistance (30, 31). Nestin^+^/CD31^+^ cells in hypoxic perivascular niche induce downstream Hes1 overexpression and promote GSLC chemotherapy resistance (32). Nestin expression is significantly elevated in drug-resistant liver cancer tissues and cell lines. Notably, nestin deficiency has been shown to reverse drug resistance, highlighting its potential as a therapeutic target to enhance the efficacy of cancer treatment. The transcriptional activation of nestin can lead to the self-protection of breast cancer cells to drugs, resulting in drug resistance (33). In addition to chemoresistance, the cells surrounding cancer cells also possess radio-resistance. In patients with brain tumors, nestin-expressing neural precursor cells (NEPs) increase the expression of IFN-γ and upregulate Shh ligands following radiation exposure, which aids in the regeneration of nerve cells by NEPs (34).

Based on these studies, inhibiting nestin can help suppress stemness, leading to reduced proliferation, invasion, and drug resistance of cancer cells. These highlights nestin as a promising therapeutic target for limiting tumor aggressiveness and improving treatment outcomes.

## Challenges associated with MMSCs in MM

The concept of MMSC has been proposed to explain the recurrence and rekindling of MM cells. Despite the near-total eradication of tumor cells in MM patients, relapses still occur (35). The status of CSCs is so crucial, but there is no consensus on the surface markers of MMSCs (**Table 1**). Currently, the mainstream is that MMSCs are located within the SP cells or ALDH1^+^ cells. However, these markers have limitations in identifying MMSCs. SP cells are not only enriched in stem cells but also include non-stem cells; meanwhile, MMSCs are not only present in the SP cells population but also exist within the main cell population (43). Additionally, bortezomib (BTZ) can significantly suppress the ratio of SP cells but increases the ratio of ALDH1^+^ cells (44, 45). SP cells and ALDH1^+^ cells may represent distinct populations. It is uncertain whether all MMSCs possess both biomarkers or if they have additional ones. These debates stem from the lack of direct evidence for the existence of a single tumor stem cell in MM tumors. Therefore, there is an urgent need to find a new marker for identifying MMSCs. Several studies have shown that nestin has been proven to be a marker for CSCs in various cancers. Nestin has been proven to be a reliable indicator for distinguishing MM patients and an important cause of 1q amplification, which can identify high risk MM. Therefore, nestin is a more specific and effective therapeutic target for MM.

**Table 1:** List of cell markers to identify MMSCs.

Population	Phenotype
Clonogenetic CD138^-^ population	CD19 ^+^ CD138^-^ (36, 37)
	CD19 ^+^ CD138^-^CD27^+^ (38)
Clonogenetic CD38^+^ population	CD38 ^+^ CD45^-^ (39)
	CD19^-^CD45^-^CD38^+^CD138^+^ (40)
	CD138^+^CD38^+^Nestin^+^
Clonogenetic CD38^-^ population	CD19 ^+^ CD38^-^CD27^+^ (41)
Clonogenetic ALDH^+^ population	CD19 ^+^ CD34^-^Lchain(λ) ^+^ ALDH^+^ (42)
	CD138^-^ALDH^+^ (38)

The development of the cutting-edge detection technologies to recognize these markers are particularly crucial. The following is an explanation of the methods for identifying MMSC markers: ①Multicolor Flow Cytometry (FCM) is a highly reliable and widely adopted clinical tool for studying cellular subpopulations in the diagnosis and treatment of MM. However, FCM data analysis requires manual gating of cell populations on two-parameter plots and is susceptible to interference between fluorescence channels. Variability in gating strategies and parameter selection among individuals significantly affects the reproducibility and stability of the data. When exploring data beyond four or five dimensions, the plotting of numerous two-dimensional graphs becomes exceedingly complex, making it difficult to represent high-dimensional data and potentially overlooking rare cell populations. Since MMSCs are often present in small quantities and easily missed, it is essential to seek new methods or technologies to address the limitations of FCM. The FlowSOM algorithm, introduced in 2018, is a rapid and precise clustering method utilizing Self-Organizing Maps (SOM) to classify all markers across all cells, with SOMs projected onto a minimum spanning tree to accentuate rare cell populations (46, 47). This method can better discover and identify rare MMSCs. ②Mass cytometry (48) is also a method used to overcome the shortcomings of commonly used FCM. Mass cytometry significantly reduces interference between channels, permitting multiparametric analysis with up to 130 distinct parameters. Although currently available reagents allow for the simultaneous measurement of up to 50 biomarkers, this method offers a new high-dimensional perspective for analyzing cellular heterogeneity, breaking the limitations of two-dimensional views (49), and enabling the high-dimensional analysis of MMSC markers. ③High-dimensional multi-pass flow cytometry based on optical barcoding significantly extends the utility of FCM from static analysis to high-throughput dynamic temporal resolution of cellular analysis. This capability allows for tracking and measuring cells over time, detecting single-cell responses to stimuli, drug treatments, or other interventions, and studying protein expression changes, including nestin, during each cell division or differentiation. It is applied in tumorigenesis and stem cell biology, breaking through the bottleneck of multi-marker analysis and capturing cell characteristics only at a single time point in FCM (50). ④Time-resolved flow cytometry can identify and quantify the up-or downregulation of key biomarkers on individual cells, analyzing the extent of expression changes in specific biomarkers on particular cells. These upregulated markers could be serve as therapeutic targets, and the downregulated markers could indicate drug resistance (51).

## Nestin and signal pathways in MM

**Figure 2:**
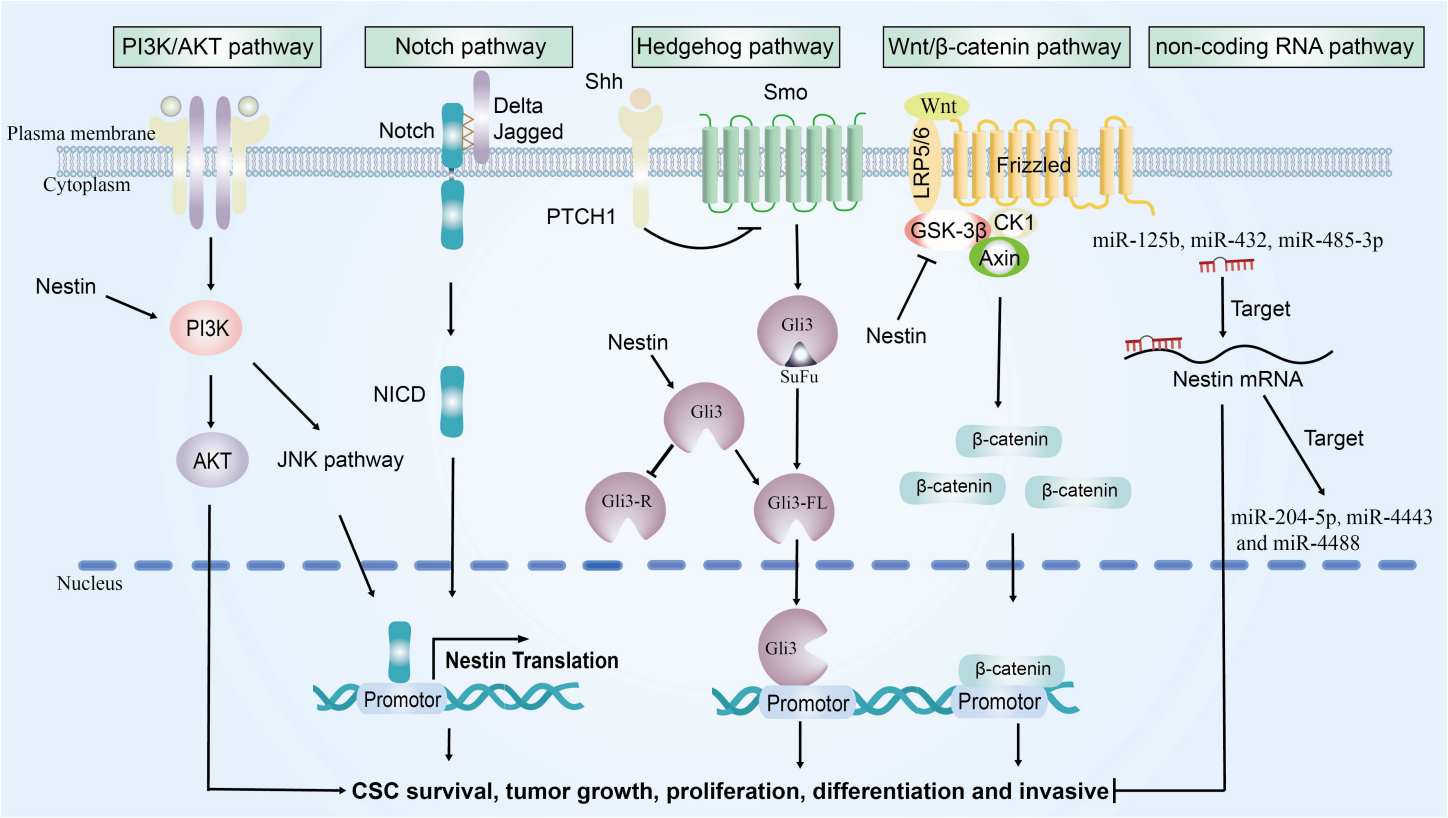
The signaling pathways network regulated by nestin. Nestin promotes tumor growth by modulating the activation of four key signaling pathways: PI3K/AKT, Notch, Hedgehog, and Wnt/β-catenin signaling pathways.

In recent years, studies have identified the expression of nestin in MM. The alterations in nestin are capable of modulating several signaling pathways. In this section, we will address the pathways that have been confirmed in other cancer types but not yet in MM, such as the PI3K/AKT, Notch, Hedgehog, and Wnt/β-catenin signaling pathway. We will also discuss other potential signaling pathways that nestin may target in MM, including the non-coding RNA pathway. In the following subsections, we will first systematically elucidate the regulatory mechanisms of nestin in each signaling pathway based on evidence from other malignancies. Subsequently, we will delve into the potential roles of these pathways in the development and progression of MM as well as their therapeutic target value. It is worth noting that these signaling pathways are also closely interconnected (**Figure 2**).

## Nestin and PI3K/AKT pathway

Studies have shown that overexpression of nestin can promote phosphorylation of PI3K (p-PI3K) and AKT (p-AKT), activating the PI3K/AKT signal pathway. Knocking down nestin reduce p-PI3K and p-AKT, leading to cell cycle arrest at G_1_, inhibiting cell proliferation, embryonic organ development, and organ size regulation (52). It should be emphasized that the activated PI3K can indirectly augment the expression of nestin via the JNK-Notch signaling cascade. This regulatory mechanism is of pivotal significance in sustaining the self-renewal and tumorigenic potential of cancer stem cells (CSCs) (53). However, another study reached the opposite conclusion. The knockdown of nestin promoted an increase in the phosphorylation of PI3K, AKT, and mTOR, enhancing the drug resistance of cancer cells (54). The reasons for the discrepancies in data are as follows: in melanoma, the depletion of nestin promotes tumor malignancy via a dual mechanism. On one hand, it upregulates the expression of matrix metalloproteinases, thereby enhancing the invasive capacity. On the other hand, it induces the aggregation of phosphorylated focal adhesion kinase on the cell membrane, which in turn promotes integrin clustering and indirectly activates the PI3K/AKT pathway, ultimately leading to the occurrence of acquired resistance (54). However, in other tumors, nestin is directly involved in the PI3K/AKT pathway. Additionally, these discrepancies may arise from differences in tumor types and cellular contexts. For instance, nestin is highly expressed in CSCs in glioblastoma, which play a crucial role in tumor initiation, maintenance, and resistance to therapy. In contrast, in melanoma, nestin expression has been primarily studied in melanoma cells rather than in CSCs.

In MM, inhibiting the PI3K/AKT/mTOR pathway suppresses the proliferation of MM side group (SP) cells, leading to cell cycle arrest, promoting apoptosis (40), inhibiting osteolytic diseases (55), and even enhancing chemotherapy sensitivity to BTZ (56), dexamethasone (57), and melphalan (58, 59), thereby inhibiting the progression of MM. Studies have shown that multiple factors can inhibit the PI3K/AKT signal pathway and inactivate the biological activity of MM. Downregulating miR-20a (60), miR-25-3p (61), and upregulating miR-30d (62), can inhibit the PI3K/AKT signal pathway, reduce the proliferation of MM or SP cells and induce apoptosis. Combination therapy of topoisomerase inhibitors with molecular inhibitors of PI3K (63), cause cell cycle arrest in MM cells. Other targeted therapeutic agents silence NUPR1 (64), DEPTOR (58), and STMN (56), inhibit the PI3K/AKT signal pathway and increase sensitivity to chemotherapy.

## Nestin and notch pathway

Research shows that nestin^+^ cells exhibit upregulation of Notch signaling (65). The Notch signal pathway can activate the nestin promoter, leading to upregulate nestin and promoting the proliferation of nestin^+^ embryonal brain tumors cells (66), and even promoting the stemness, proliferation, and impaired differentiation of glioma stem cells (67). Furthermore, in the absence of p53, the dedifferentiation of mature liver cells into nestin^+^ progenitor-like cells is promoted, accompanied by the activation of the intracellular Notch signal pathway (68).

In MM, as the disease progresses, there is a continuous overexpression of Notch-1 and nestin (69, 70). Additionally, activation of the Notch signal pathway promotes the upregulation of Notch, Jagged1, and Hes1, enhancing MM proliferation, inhibiting apoptosis, and increasing drug resistance (70). Inhibition of the Notch signal pathway can lead to cell cycle arrest and ameliorate cancer-induced bone destruction (71). Overall, in MM, there is a potential correlation between the upregulation of Notch signaling and the increased expression of nestin. The cell cycle regulator GANT61 inhibits the Notch signal pathway, suppresses MM cell proliferation, promotes apoptosis, and leads to G_1_/G_0_ cycle arrest (72).

## Nestin and Hedgehog pathway

Nestin primarily activates the Hedgehog (Hh) signal pathway through a Smoothened (Smo)-independent manner to cause genomic instability and facilitate tumorigenesis (73). The overexpression of nestin augments the levels of Gli3FL (the full-length form of Gli3) while diminishing the levels of Gli3R (the repressive form of Gli3), thereby enhancing the activity of the Hh signaling pathway through the inhibition of Gli3 phosphorylation (74). Targeting nestin can restore Gli3 function and inhibit tumor cell proliferation (74, 75). Meanwhile, the Hh signaling can regulate nestin expression. Hh signaling induces the upregulation of Gli1, promoting the expression of nestin in both cancer stem cells (76) and neural progenitor cells (77). Additionally, the Shh signaling can drive nestin expression through Gli1-independent mechanism, promoting the proliferation of nestin^+^ precursor cells (34). Introducing RCAS vectors expressing SHh and c-Myc into nestin^+^ progenitor cells can induce proliferation and transformation into malignant tumor cells, leading to tumorigenesis (78).

In MM, Hh signaling activity can be activated both through the canonical pathway (Smo-dependent) and Smo-independent mechanisms. MM cell lines activate the SHh/Gli1 axis through autocrine SHh, promoting the expression of SHH and Gli1, thereby enhancing proliferation and protecting myeloma cells from spontaneous and stress-induced apoptosis (79). Additionally, Hh signaling in CD138^−^ MMSC is activated, promoting the clonal expansion of MMSC, sustaining the MM tumor stem cell niche (80), and conferring BTZ resistance (81). Hh inhibitors mainly inhibit Hh signaling by binding to Smo. Liver X receptor activators inhibit Hh pathway activity *in vitro*, thereby suppressing the growth of clonal tumors *in vitro* and MM stem cells *in vivo*, leading to the loss of tumor initiation and self-renewal potential (82). In addition, overexpression of miR-324-5p (81) and miR-1271 (83) significantly reduced the expression of Hh signal components Smo and Gli1, inhibiting the growth, survival, and stem cell compartment of MM cells, and enhancing sensitivity to BTZ.

## Nestin and Wnt/β-catenin pathway

Nestin overexpression enhances the activity of the Wnt/β-catenin signal pathway, reducing levels of GSK-3β and promoting β-catenin nuclear localization, thereby improving tumor proliferation, invasion, and metastasis, and even reversing drug resistance, providing new strategies for cancer treatment (84). Additionally, inhibiting nestin in breast cancer stem cells could increases GSK-3β and E-cadherin, decreases β-catenin, N-cadherin and vimentin to reduce proliferation and invasion of CSCs (85). Furthermore, Wnt signaling can induce nestin expression by increasing β-catenin protein, leading to excessive proliferation or dedifferentiation of donor cells and promoting tumor formation (86).

In MM, the abnormal activation of Wnt/β-catenin signaling is involved in the pathogenesis of MM, particularly in maintaining the stemness of MMSCs (87). Promoting the proteasomal degradation of β-catenin through GSK-3β-independent mechanism can inhibit the Wnt/β-catenin signal pathway, preventing the proliferation of MM cells (88, 89) and even reducing the population of ALDH1^+^ MMSCs (44). Furthermore, Wnt inhibitors reduce β-catenin expression, suppress the anti-proliferative activity of MM cells against dexamethasone (90), BTZ (91), and lenalidomide (92), and inhibit the progression of MM. The combination of TNKSi and PORCi (91), picriol (93), and pyrvinium pamoate (94) exerts the same effect on MM by inhibiting the Wnt/β-catenin pathway. Overexpression of miR-744-5p (95), miR-19a-3p (96), and miR-30-5p (97) also inhibit the Wnt/β-catenin signal pathway to suppress the proliferation of MM cells and promote apoptosis.

## Nestin and non-coding RNA

Several miRNAs, such as miR-125b (98), miR-432 (99), and miR-485-3p (100), have been shown to target and suppress nestin expression. High expression levels of miR-21 correlate positively with nestin mRNA in Meningiomas (101), and suppression of miR-21 can reduce nestin expression, leading to decreased melanoma tumor growth (102).

In MM, overexpression of miR-21 is associated with disease resistance. Downregulation of miR-21 exhibits anti-MM activity by inhibiting the growth of MM cells both *in vitro* and *in vivo* (103). MiR-21 can also impair the differentiation of Th17 cells, abrogating Th17-mediated MM cells proliferation and osteoclast activity (104). Overexpression of miR-125b-5p can trigger apoptosis and autophagy in MM cells, suppressing tumor activity (105). Clinically, miR-21 and miR-125b-5p are closely related to the treatment or prognosis of MM. Prior to lenalidomide-dexamethasone treatment/diagnosis, ratios of both miR-16 and miR-21 expression levels below two predicted a shorter time to response and a longer time to next treatment (106). Plasma levels of miR-125b-5p in MM are correlated with clinical stage and progression-free survival of patients (107). Nestin is also a direct target of several miRNAs, including miR-204-5p (108), miR-4443, and miR-4488 (109). In addition to miRNA, circular RNAs (circRNAs) such as circTTC3 can upregulate the expression of nestin, thereby promoting the proliferation of neural stem cells (110).

## The clinical relevance of nestin in MM

In 2011, the Svachova team firstly detected nestin in mature CD138^+^/CD38^+^ plasma cell (PC) of MM patients, confirming that nestin is a tumor specific marker of CD138^+^/CD38^+^ PC in MM patients (111). Further, another study analyzed the nestin expression across a spectrum of individuals, ranging from those without hematological malignancies, through monoclonal gammopathy of undetermined significance (MGUS) and MM patients, to plasma cell leukemia and MM cell lines (111). Nestin can be evaluated as a reliable marker for accurately distinguishing MM patients from the control group, and its expression gradually increases as the disease progresses (41). Additionally, the Svachova team found that nestin is located on chromosome 1q and is an important cause of 1q amplification. 1q amplification was classified as a high-risk cytogenetic anomaly in the mSMART and R2-ISS staging system. The latest R2-ISS is the second revised version of the ISS updated by the European Myeloma Network (EMN) in 2022. Compared with R-ISS, R2-ISS is an improved and simple state-of-the-art staging system that adds 1q amplification in scoring, resulting in better stratification of especially the large group of patients with intermediate-risk newly diagnosed MM (112). 1q amplification has been shown to be a poor prognostic factor (112), associated with recurrent/refractory MM (RRMM), and increases the risk of MM progression (113). Therefore, there is a strong correlation between nestin and clinical staging of MM.

There have been studies exploring the treatment targeting nestin. The knockdown of nestin via short hairpin RNA (shRNA) or small interfering RNA (siRNA) techniques can significantly inhibit cell proliferation, migration, and invasion. Lu et al. successfully suppressed nestin expression using shRNA, which notably decelerated the growth of glioblastoma (114). Moreover, knockout of the nestin gene via CRISPR/Cas9 technology completely abolishes nestin expression (54). Certain small-molecule compounds, such as resveratrol, have been demonstrated to suppress nestin expression (89). Resveratrol, a plant antitoxin, inhibits the growth, migration, invasion, and expression of nestin in glioblastoma cells (115). Given that nestin-positive CSCs can evade host immune surveillance through PD-L1 expression (116, 117), anti-PD-1/PD-L1 therapy may be effective for nestin-positive MM. In addition, due to the role of nestin regulating the cytoskeleton, anti-microtubule inhibitors such as cytorelaxin D and zeaxanthin A have been shown to have anti-nestin activity (28). Furthermore, all trans retinoic acid (ATRA) reduces the expression of nestin in CSCs, inhibits CSCs proliferation, and is even associated with the PI3K/AKT/mTOR and Wnt/β-catenin pathways (118, 119). Another study found that arsenic trioxide inhibits the levels of Notch1 and Hes1 proteins, reduces nestin^+^ cells, and thus decreases the CSC populations in gliomas (120). Nitidine chloride can inhibit the expression of nestin, a tumor CSC related factor, through the Hedgehog pathway. At present, the regulation of oxidative stress is becoming a cancer treatment method. In MM, it is manifested as excessive production of ROS, and nestin has been found to promote cancer cell antioxidant activity, thereby promoting proliferation, invasion, and drug resistance. Therefore, regulating oxidative stress status can be a potential therapeutic method for anti-nestin (121). Based on the above findings, multiple anti-tumor approaches affect the expression of nestin through various pathways, thereby influencing the biological activity of CSCs.

However, the therapeutic strategies targeting nestin currently face significant challenges: existing therapies are confined to directly or indirectly modulating nestin expression via inhibitors, and these approaches have only been validated in other malignancies but not yet in MM. To surmount the existing limitations, future research should focus on validating these approaches in MM and developing innovative therapies, including: developing specific CAR-T cell therapies targeting nestin; and advancing the clinical translation of nestin inhibitors to transcend the constraints of current laboratory-based research. It is noteworthy that nestin is also basally expressed in specific progenitor and stem cell populations in normal tissues. Studies have shown that peptides can bind to the N-terminal isoform of nestin specifically expressed in glioma stem cells, a property that enables them to target nestin-positive cell populations in human glioma tissues (122). Future research should be focused on developing efficient and specific tumor-targeting delivery systems for the precise delivery of anti-nestin therapeutic agents. Additionally, a systematic evaluation of the side effects of these therapeutic approaches on normal stem cell populations expressing nestin is warranted.

The clinical treatment of inhibiting the MM signal pathway through drugs is currently under investigation. Inhibitors of these signal pathways may affect the expression of nestin. The safety, tolerability, and preliminary activity of CUDC-907 (the first oral dual inhibitor of HDAC and PI3K) in RRMM patients have been tested in phase I trials (123) In addition, two inhibitors of Hedgehog, vismodegib and sonidegib, have been approved by the US Food and Drug Administration (FDA) (124). Sonidegib, an oral small molecule SMO receptor antagonist, used for the treatment of cancer and is currently undergoing phase I/II studies on other malignant tumors, including MM (125). Bisphosphonate-conjugated γ-secretase inhibitors (BT-GSI), as a type of Notch inhibitor targeting the bone/bone marrow microenvironment, are capable of simultaneously inhibiting the growth of MM cells and preventing bone loss, while exhibiting safer therapeutic characteristics (126). Additionally, DKK1, as a canonical antagonist of the WNT/β-catenin pathway, plays a pivotal role in the process of bone destruction in MM. BHQ-880, a monoclonal antibody targeting DKK1, completed a Phase II clinical trial for multiple myeloma in 2020. The trial results demonstrated that BHQ-880 significantly reduced the extent of bone destruction in MM patients and exhibited good tolerability during the treatment process (127). Therefore, it is feasible to use anti-tumor drugs targeting the signal pathway to target nestin for the treatment of MM in future research.

In summary, nestin is a core factor in 1q amplification, and can serve as a reliable biomarker for distinguishing high-risk MM. A variety of clinical drugs affect the expression of nestin through signal pathways, and then affect the proliferation of CSCs and myeloma. Nestin is a potential therapeutic target for MM.

## Conclusions and perspectives

In this review, we summarized that nestin is a potential therapeutic target for MMSC. Firstly, we described the characteristics of nestin, including its gene structure and protein features. The role of nestin in CSCs of solid tumors were summarized. Subsequently, we explored the advantages and disadvantages of current MMSC markers, as well as potential directions for detection technologies. Nestin interactions with several oncogenic stemness pathways in MMSCs are the core. For the clinical value, nestin serves as a reliable biomarker for identifying high-risk MM patients who are likely to exhibit resistance to standard treatments. Finally, we discussed the potential of nestin as a target for therapeutic intervention through the inhibition of the aforementioned signaling pathways, as well as the challenges faced in targeting nestin.

While research on nestin in MM is not yet comprehensive, future studies can further validate whether targeting nestin can eliminate MMSCs and weak MM progression. Larger-scale and higher-quality systematic reviews and meta-analyses are needed to enhance the accuracy of nestin in MM stratification, as well as prognostic assessment. Additionally, RRMM poses a significant challenge in MM treatment. Nestin is an important factor in 1q amplification, indicating that it may be a predictive molecule for RRMM. Exploring small molecule drugs targeting nestin or its regulatory network using the Perturb-DBiT database can provide a comprehensive, spatially resolved view of perturbation responses in complex tissues, thereby leading to the development of new therapeutic strategies (128–130). In the future, the application of the co-indexing of transcriptomes and epitopes (CITE) technology holds the potential to deeply characterize the proteomic and whole-transcriptomic profiles in patients with MM, thereby enabling precise evaluation of nestin gene expression levels (131). In all, the review’s synthesis of current knowledge underscores the clinical relevance of nestin and highlights important areas for future research, not only as a prognostic indicator but also as a potential target for innovative therapies aimed at overcoming treatment resistance in MM.
